# Comparative karyotype study of three Cyprinids (Cyprinidae, Cyprininae) in Thailand by classical cytogenetic and FISH techniques

**DOI:** 10.3897/CompCytogen.v14i4.54428

**Published:** 2020-12-22

**Authors:** Sumalee Phimphan, Patcharaporn Chaiyasan, Chatmongkon Suwannapoom, Montri Reungsing, Sippakorn Juntaree, Alongklod Tanomtong, Weerayuth Supiwong

**Affiliations:** 1 Biology program, Faculty of Science and Technology, Phetchabun Rajabhat University, Phetchabun 67000, Thailand Phetchabun Rajabhat University Phetchabun Thailand; 2 Toxic Substances in Livestock and Aquatic Animals Research Group, Department of Biology, Faculty of Science, Khon Kaen University, Muang, Khon Kaen 40002, Thailand Khon Kaen University Khon Kaen Thailand; 3 Department of Fishery, School of Agriculture and Natural Resources, University of Phayao, Muang, Phayao 56000, Thailand University of Phayao Phayao Thailand; 4 Department of Biotechnology, Faculty of Science and Technology, Rajamangala University of Technology Tawan-ok, Sri Racha, Chon Buri 20110, Thailand Rajamangala University Chon Buri Thailand; 5 Faculty of Interdisciplinary Studies, Nong Khai Campus, Khon Kaen, University, Muang, Nong Khai 43000, Thailand Khon Kaen University Nong Khai Thailand

**Keywords:** Chromosome, *Epalzeorhynchos frenatum*, FISH, *Puntigrus partipentazona*, *Scaphognathops bandanensis*

## Abstract

Three species of ornamental fishes in the subfamily Cyprininae (family Cyprinidae) namely, *Epalzeorhynchos frenatum* (Fowler, 1934), *Puntigrus partipentazona* (Fowler, 1934), *Scaphognathops bandanensis* Boonyaratpalin et Srirungroj, 1971 were studied by classical cytogenetic and fluorescent in situ hybridization (FISH) techniques. Chromosomes were directly prepared from kidney tissues and stained by using conventional and Ag-NOR banding techniques. Microsatellite d(CA)_15_ and d(CGG)_10_ probes were hybridized to the chromosomes of three cyprinids. The results show that the three cyprinid species share the same diploid number as 2n=50 but there are differences in the fundamental number (NF) and karyotypes i.e. *E. frenatum*: NF = 78, 18m+10sm+10st+12a; *P. partipentazona*: NF = 80, 6m+24sm+14st+6a; *S. bandanensis*: NF = 66, 4m+12sm+34a. NOR positive masks were observed at the regions adjacent to the telomere of the short arm of the chromosome pairs 10 (submetacentric) and 1 (metacentric) in *E. frenatum* and *P. partipentazona*, respectively whereas those were revealed at telomeric regions of the long arm of the chromosome pair 9 (acrocentric) in *S. bandanensis*. The mapping of d(CA)_15_ and d(CGG)_10_ microsatellites shown that hybridization signals are abundantly distributed in telomeric regions of several pairs except d(CA)_15_ repeats in *S. bandanensis*, which are distributed throughout all chromosomes and d(CGG)_10_ repeats in *P. partipentazona* display the high accumulation only in the first chromosome pair.

## Introduction

There are about 200 species of freshwater fish used as ornamentals in Thailand. More than half of all ornamental fishes in Thailand belong to the family Cyprinidae. The most popular species include *Betta splendens* Regan, 1910, *Gyrinocheilus aymonieri* (Tirant, 1883), *Epalzeorhynchos bicolor* (Smith, 1931), *E. frenatum* (Fowler, 1934), *Puntigrus tetrazona* (Bleeker, 1855), *Channa micropeltes* (Cuvier, 1831), *Barbonymus alter* Bleeker, 1853, *Bar. schwanenfeldii* (Bleeker, 1854) and *Balantiocheilos melanopterus* (Bleeker, 1850) (Sermwatanakul 2005).

**Figure 1.**
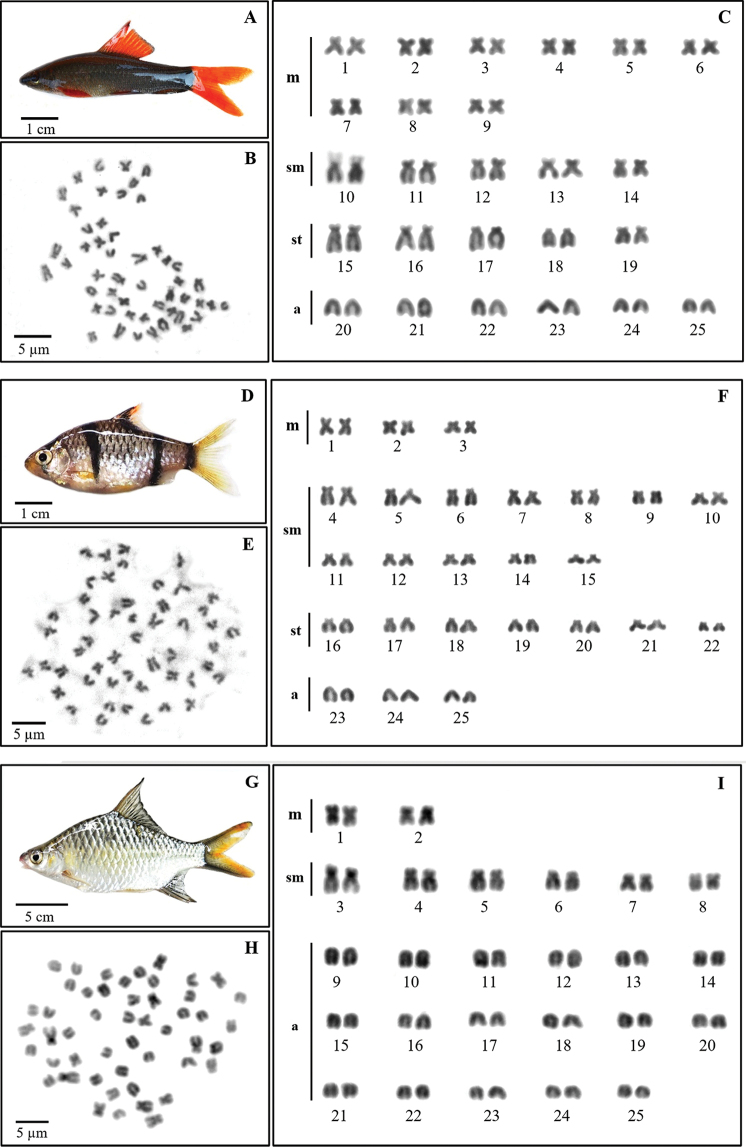
Specimens, metaphase chromosome plates and karyotypes of *Epalzeorhynchos frenatum* (**A–C**), *Puntigrus partipentazona* (**D–F**), *Scaphognathops bandanensis* (**G–I**) by conventional technique.

Family Cyprinidae is the most abundant and globally widespread family of freshwater fish, comprising 3,000 extant and extinct species in about 370 genera (Eschmeyer et al. 2015). The subfamily Cyprininae is one of the largest groups of this family. The essential large tribes such as Labeonini, Poropuntiini and Smiliogastrini have many species that are economically important ornamental fish of Thailand, namely *Epalzeorhynchos frenatum* (Fowler, 1934), *Puntigrus partipentazona* (Fowler, 1934), *Scaphognathops bandanensis* Boonyaratpalin et Srirungroj, 1971 (Fig. 1A, D, G). However, there are few studies of cytogenetics of these ornamental fishes. To date, most reports are of conventional technique studies to determine chromosome number and karyotype composition and only a few ionclude NOR banding analysis. The 2n ranges from 48–50 in the tribes Labeonini and Smiliogastrini while the tribe Poropuntiini is more conserved as 2n = 50 (Arai 2011) (Table 1). Understanding of the basic information on cytogenetics can be applied to the development of potentially commercial stains/species in the future. The studies on the karyotypes help to investigate the genetic structure of aquatic animal species in each habitat, thus it can determine what species are related to each other in an accurate manner. This may help to facilitate the hybridization between them in the future for strain improvement (Sofy et al. 2008), breeding practices of organisms by using chromosome set management (Na-Nakorn et al. 1980), brood stock selection (Mengampan et al. 2004).

**Table 1.** Reviews of cytogenetic reports in the tribes Labeonini, Poropuntiini, and Smiliogastrini. (2n = diploid number, m = metacentric, sm = submetacentric, st = subtelocentric, a = acrocentric and NORs = nucleolar organizer regions, NF = fundamental number, – = not available).

Tribe / Genus / Species	2n	NF	Formula	NORs	Reference
Tribe Labeonini					
*Barbichthys laevis* (Valenciennes, 1842)	50	76	20m+6sm+4st+20a	–	Donsakul et al. (2006)
*Bangana devdevi* (Hora, 1936)	50	86	20m+16sm+14a	–	Donsakul et al. (2011)
*Cirrhinus julleini*	50	90	26m+14sm+4st+6a	–	Magtoon and Arai (1993)
(Valenciennes, 1844)	50	92	36m+6sm+2st+6a	–	Donsakul (1997)
*C. microlepis* Sauvage, 1878	50	88	22m+8sm+8st+12a	–	Donsakul and Magtoon (1997)
	50	72	12m+10sm+2st+26a	–	Donsakul et al. (2007)
*Epalzeorhynchos frenatum* (Fowler, 1934)	48	72	14m+10sm+8st+16a	–	Donsakul and Magtoon (1993)
	**50**	**78**	**18m+10sm+10st+12a**	2	**Present study**
*E. bicolor* (Smith, 1931)	50	74	20m+4sm+2st+24a	–	Donsakul and Magtoon (1993)
*E. munensis* (Smith, 1934)	50	84	22m+12sm+2st+14a	–	Donsakul et al. (2012)
*Garra cambodgiensis* (Tirant, 1883)	50	82	20m+12sm+4st+14t	–	Donsakul et al. (2016)
*G. fasciacauda* Fowler, 1937	50	84	18m+14sm+2st+16t	–	Donsakul et al. (2016)
*G. notata* (Blyth, 1860)	50	80	20m+10sm+20t	–	Donsakul et al. (2016)
*Incisilabeo behri* (Fowler, 1937)	50	78	12m+16sm+4st+18t	–	Donsakul and Magtoon (2003)
*Labeo chrysophekadian* (Bleeker, 1850)	50	78	4m+10sm+14st+22a	–	Seetapan (2007)
*Labiobarbus lineatus* (Sauvage, 1878)	50	80	20m+10sm+20a	–	Magtoon and Arai (1990)
*L. spiropleura* (Sauvage, 1881)	50	90	34m+4sm+2st+10a	–	Donsakul and Magtoon (1997)
*Mekongina erythrospila* Fowler, 1937	50	74	10m+14sm+26a(t)	–	Donsakul and Magtoon (2003)
*Osteochilus melanopleura* (Bleeker, 1852)	50	96	36m+10sm+2st+2a	–	Donsakul and Magtoon (1995)
*O. microcephalus* (Valenciennes, 1842)	50	86	26m+10sm+14st	–	Donsakul et al. (2001)
*O. vittatus* (Valenciennes, 1842)	50	96	16m+30sm+4st	–	Magtoon and Arai (1990)
	50	86	26m+10sm+14st	–	Donsakul (1997)
*O. waandersi* (Bleeker, 1853)	50	92	18m+24sm+4st+4a	2	Magtoon and Arai (1993)
*Puntioplites falcifer* Smith, 1929	50	80	14m+16sm+2st+18a	–	Donsakul et al. (2007)
	50	92	16m+10sm+16a+8t	–	Sophawanus et al. (2017)
Tribe Smiliogastrini					
*Osteobrama alfrediana* (Valenciennes, 1844)	50	96	24m+22sm+4a	–	Donsakul et al. (2011)
*Hampala disper* Smith, 1934	50	70	5m+5sm+3st+12a	–	Donsakul and Poopitayasathaporn (2002)
*H. macrolepidota* Kuhl & Van Hasselt, 1823	50	72	10m+12sm+8st+20a	–	Donsakul and Poopitayasathaporn (2002)
*Puntigrus partipentazona* (Fowler, 1934)	50	90	6m+34sm+10a	–	Taki et al. (1977)
	**50**	**80**	**6m+24sm+14st+6a**	**2**	**Present study**
*P. tetrazona* (Bleeker, 1855)	50	84	34m+6st+10a	–	Ohno et al. (1967)
	50	84	6m+28sm+16a	–	Hinegardner and Rosen (1972), Taki et al. (1977), Suzuki et al. (1995)
	50	–	–	–	Krishnaja and Rege (1980) Vinogradov (1998)
*P. tetrazona partipentazona* (Fowler, 1937)	50	90	6m+34sm+10a	–	Taki et al. (1977)
*Puntius arulius* (Jerdon, 1849)	50	82	6m+26sm+18a	–	Taki and Suzuki (1977)
	50	90	10m+18sm+12st+10t	–	Arunachalan and Murugan (2007)
*P. binotatus* (Valenciennes, 1842)	50	92	8m+34sm+8a	–	Taki et al. (1977)
*P. brevis* (Bleeker, 1850)	50	70	6m+14sm+8st+22a	–	Khuda-Bukhsh (1975)
	50	54	2m+2sm+2st+22a	–	Donsakul and Poopitayasathaporn (2002)
	48	56	2m+6st+40a	–	Seetapan (2007)
	50	62	4m+4sm+4a+38t	2	Nitikulworawong and Khrueanet (2014)
*P. chola* (Hamilton, 1822)	50	56	2m+4sm+44a	–	Taki and Suzuki (1977)
	50	54	2m+2sm+4st+42a	–	Tripathi and Sharma (1987)
	50	54	2m+2sm+46a	–	Sahoo et al. (2007)
*P. conchonius* (Hamilton, 1822)	50	94	6m+38sm+6a	–	Hinegardner and Rosen (1972), Taki and Suzuki (1977)
	48	78	10m+20sm+10st+8a	–	Sharma and Agarwal (1981)
	50	–	–	–	Vasiliev (1985)
	50	90	16m+24sm+2st+8a	–	Khuda et al. (1986), Ojima and Yamamoto (1990)
*P. conchonius* (Hamilton, 1822)	50	94	4m+40sm+6a	–	Takai and Ojima (1988)
*P. cumingi* (Günther, 1868)	50	94	18m+26sm+6a	–	Taki and Suzuki (1977)
*P. daruphani* Smith, 1934	50	70	12m+8sm+6st+24a	–	Magtoon and Arai (1989)
*P. denisonii* (Day, 1865)	50	74	4m+20sm+18st+8a	8	Nagpure et al. (2004)
*P. everetti* (Boulenger, 1894)	50	86	6m+30sm+14a	–	Hinegardner and Rosen (1972), Taki et al. (1977), Vinogradov (1998)
*P. fasciatus* (Jerdon, 1849)	50	80	30m+4st+16a	–	Ohno et al. (1967)
	50	82	6m+26sm+18a	–	Taki et al. (1977)
*P. filamentosus* (Valenciennes, 1844)	50	84	8m+26sm+16a	–	Taki and Suzuki (1977)
	50	78	12m+16sm+12st+10a	8	Nagpure et al. (2003)
*P. lateristriga* (Valenciennes, 1842)	50	88	6m+32sm+12a	–	Taki et al. (1977)
	50	86	22m+14sm+6st+8a	–	Sobita et al. (2004)
*P. melanampyx* Day, 1865	50	74	12m+12sm+14st+12a	–	Khuda et al. (1986)
*P. nigrofasciatus* (Günther, 1868)	50	100	16m+34sm	–	Taki and Suzuki (1977)
*P. oligolepis* (Bleeker, 1853)	50	88	8m+30sm+12a	–	Taki et al. (1977)
	50	80	14m+16sm+4st+16a	–	Arai and Magtoon (1991)
	50	92	6m+36sm+8a	–	Taki et al. (1977)
*P. pentazona* (Boulenger, 1894)	50	98	22m+26sm+2a	–	Taki et al. (1977)
*P. sarana* (Hamilton, 1822)	50	76	12m+14sm+12st+12a	–	Rishi (1981)
*P. sarana subnasutus* (Valenciennes, 1842)	50	88	12m+26sm+8st+4a	–	Nagpure et al. (2004)
*P. semifasciolatus* (Günther, 1868)	50	76	12m+14sm+14st+10a	–	Gui et al. (1986), Yu et al. (1989)
	50	76	12m+14sm+14st+10a	8	Nagpure et al. (2004)
	50	76	8m+18sm+24a	–	Suzuki (1991)
*P. sophore* (Hamilton, 1822)	48	52	2m+2sm+44a	–	Rishi (1973)
	48	54	2m+4sm+42a	–	Rishi et al. (1977)
	48	52	4m+2st+42a	–	Rishi and Rishi (1981)
	50	56	2m+4sm+44a	–	Khuda et al. (1986)
	48	52	4m+6st+38a	–	Tripathi and Sharma (1987)
*P. sophoroides* (Günther, 1868)	50	54	2m+2sm+46a	–	Magtoon and Arai (1989)
*P. stoliczkanus* (Day, 1871)	50	94	22m+22sm+4st+2a	–	Magtoon and Arai (1989)
*P. tambraparniei* Silas, 1954	50	94	12m+16sm+16a+6t	–	Arunachalan and Murugan (2007)
*P. ticto* (Hamilton, 1822)	50	82	20m+12sm+10st+8a	–	Sharma et al. (1995), Vinogradov (1998)
	50	100	28m+22sm	–	Taki and Suzuki (1977)
	50	94	28m+16sm+6st	–	Sahoo et al. (2007)
*P. titteya* (Deraniyagala, 1929)	50	98	20m+28sm+2a	–	Hinegardner and Rosen (1972), Taki and Suzuki (1977)
	48	52	4m+2sm+42a	–	Khuda-Bukhsh and Barat (1987)
*Systomus* sp.1	50	82	12m+20sm+6st+12a	–	Donsakul et al. (2006)
*S. binotatus* (Valenciennes, 1842)	50	88	24m+14sm+12a	–	Donsakul and Magtoon (2002)
*S. orphoides* (Valenciennes, 1842)	50	82	12m+20sm+4st+14a	–	Piyapong (1999)
	50	74	8m+16sm+10st+16a	–	Donsakul and Poopitayasathaporn (2002)
*S. stoliczkanus* (Day, 1871)	50	94	24m+20sm+6a	–	Donsakul et al. (2011)
Tribe Poropuntiini					
*Amblyrhynchichthys truncatus* (Bleeker, 1851)	50	78	16m+12sm+22a	–	Donsakul et al. (2006)
*Balantiocheilos melanopterus* (Bleeker, 1850)	50	72	10m+12sm+28a	–	Ojima and Yamamoto (1990)
	50	70	14m+6sm+10st+20a	–	Donsakul and Poopitayasathaporn (2002)
*Barbonymus gonionotus* (Bleeker, 1850)	50	72	2m+20sm+4st+24a	–	Magtoon and Arai (1989)
	50	74	16m+8sm+26a	–	Donsakul and Magtoon (1997)
	50	72	6m+16sm+6st+22a	–	Piyapong (1999)
	50	66	2m+4sm+10st+34a	–	Seetapan (2007)
	50	74	6m+18sm+16st+10a	2	Khuda-Bukhsh and Das (2007)
*Cosmochilus harmandi* Sauvage, 1878	50	82	22m+10sm+10st+8a	–	Donsakul et al. (2005)
*Cyclocheilichthys apogon* (Valenciennes, 1842)	50	70	12m+8sm+6st+24a	–	Magtoon and Arai (1989)
	50	76	18m+8sm+4st+20a	–	Donsakul and Poopitayasathaporn (2002)
	50	86	10m+16sm+10a+14t	6	Chantapan (2015)
*C. lagleri* Sontirat, 1989	50	86	12m+6sm+1st+6a	–	Donsakul et al. (2006)
*C. repasson* (Bleeker, 1851)	50	78	12m+16sm+6st+16a	–	Donsakul et al. (2005)
	50	84	6m+6sm+22st+16a	–	Seetapan (2007)
*Cyclocheilos enoplos* (Bleeker, 1849)	50	90	10m+30sm+4st+6a	4	Magtoon and Arai (1993)
	50	72	14m+8sm+10st+18a	–	Donsakul and Magtoon (1995a)
	50	78	16m+12sm+6st+16a	–	Donsakul and Poopitayasathaporn (2002)
*Hypsibarbus lagleri* Rainboth, 1996	50	74	4m+20sm+26a	–	Donsakul and Magtoon (2001)
*H. malcolmi* (Smith, 1945)	50	64	10m+4sm+36a	–	Donsakul et al. (2007)
*H. vernayi* (Norman, 1925)	50	58	6m+2sm+4st+38a	–	Donsakul and Magtoon (2002)
*H. wetmorei* (Smith, 1931)	50	70	12m+8sm+6st+24a	–	Magtoon and Arai (1989)
	50	74	12m+12sm+4st+22a	2	Piyapong (1999)
	50	74	12m+12sm+2st+24a	–	Donsakul and Magtoon (2002)
	50	82	10m+14sm+8a+18t	6	Chantapan (2015)
*Mystacoleucus argenteus* (Day, 1888)	50	76	6m+20sm+2st+22a	–	Donsakul et al. (2006)
*M. marginatus* (Valenciennes, 1842)	50	76	16m+10sm+24a	–	Arai and Magtoon (1991)
	50	68	14m+4sm+2st+30a	–	Donsakul and Poopitayasathaporn (2002)
*Poropuntius deauratus* (Valenciennes, 1842)	50	74	14m+10sm+26t	–	Donsakul et al. (2005)
*P. sinensis* (Bleeker, 1871)	50	82	10m+22sm+18st	–	Zen et al. (1984)
*P. laoensis* (Günther, 1868)	50	74	14m+10sm+10st+16a	–	Donsakul and Magtoon (2008)
*P. normani* Smith, 1931	50	72	10m+12sm+28a	–	Donsakul et al. (2007)
*P. chonglingchungi* (Tchang, 1938)	50	80	12m+18sm+20st	–	Zen et al. (1986)
*Scaphognathops bandanensis* Boonyaratpalin & Srirungroj, 1971	50	66	10m+6sm+34a	–	Donsakul et al. (2007)
	**50**	**66**	**10m+6sm+34a**	**2**	**Present study**
*Sikukia gudgeri* (Smith, 1934)	50	68	10m+8sm+4st+28a	–	Donsakul et al. (2005)

For some species, the simple characterization of the karyotype may be sufficient to identify intra- and inter-specific variants. However, in most cases, just the karyotype description appears to be inconclusive when not coupled with other methods capable of generating more accurate chromosomal markers. In this sense, the use of molecular cytogenetic analyses has played an important role in the precise characterization of the structure of genomes (Cioffi and Bertollo 2012). Multiple DNA copies or repetitive DNAs are a large substantial portion of the genome of eukaryotes that can be generally classified into two main classes: tandem repeats, such as the multigene families and the satellite DNAs; and the dispersed elements, such as transposons and retrotransposons, known as Transposable elements (TEs) (Jurka et al. 2005). Among the tandem repeats we can find the highly-repeated satellite DNAs and “moderate repeats”, like mini- and microsatellite DNA (Charlesworth et al. 1994). These non-coding DNA sequences are organized as long arrays of head-to-tail linked repeats (Plohl et al. 2008).

Recently, the molecular cytogenetic studies using fluorescence *in situ* hybridization (FISH) for mapping repetitive DNA sequences have provided important contributions to the characterization of the biodiversity and the evolution of divergent fish groups (Cioffi and Bertollo 2012). Moreover, some microsatellite repeats are species-specific characters among some fish group (Cioffi et al. 2015). Most molecular cytogenetic studies in cypinid fishes were performed by FISH technique using rDNA probes (Inafuku et al. 2000; Kikuma et al. 2000; Ocalewicz et al. 2004; Zhu et al. 2006; Singh et al. 2009; Rossi et al. 2012; Nabais et al. 2013; Kirtiklis et al. 2014; Spoz et al. 2014; Han et al. 2015; Kumar et al. 2016; Han et al. 2017). However, NOR banding including fluorescence *in situ* hybridization (FISH) techniques to investigate chromosomal distribution of repetitive DNA sequences on the chromosomes of *E. frenatum*, *P. partipentazona*, *S. bandanensis* have not been performed.

In present study, we carried out an analysis of chromosomal structures and genetic markers on *E. frenatum*, *P. partipentazona*, and *S. bandanensis* using cytogenetics, and molecular cytogenetics techniques. The knowledge revealed will provide a powerful tool for the next generation of genome research in Thai freshwater fishes and discovering biodiversity, with useful applications in fish breeding for conservation and commercials of ornamental species. Moreover, it is useful applications in evolution, systematics, phylogenetics, fish fauna management and suitable conservation of river basin.

## Material and methods

Ten males and ten females of each species including *E. frenatum*, *P. partipentazona*, *S. bandanensis*, were collected from the Song Khram, Chi and Mekong Basins, respectively. Preparation of fish chromosomes was from kidney cells (Pinthong et al. 2015; Supiwong et al. 2015). The chromosomes were stained with Giemsa’s solution for 10 min. Ag-NOR banding was performed by applying two drops of 2% gelatin on the slides, followed with four drops of 50% silver nitrate (Howell and Black 1980). Metaphase figures were analyzed according to the chromosome classification of Levan et al. (1964). Chromosomes were classified as metacentric (m), submetacentric (sm), subtelocentric (st) or acrocentric (a). Fundamental number, NF (number of chromosome arm) is obtained by assigning a value of two to metacentric and submetacentric chromosomes and one to subtelocentric and acrocentric chromosomes.

The use of microsatellite d(CA)_15_ and d(CGG)_10_ probes described by Kubat et al. (2008) was followed here with slight modifications. These sequences were directly labeled with Cy3 at 5´ terminal during synthesis by Sigma (St. Louis, MO, USA). FISH was performed under high stringency conditions on mitotic chromosome spreads (Pinkel et al. 1986). After denaturation of chromosomal DNA in 70% formamide/ 2×SSC at 70 °C, spreads were incubated in 2×SSC for 4 min at 70 °C. The hybridization mixture (2.5 ng/µL probes, 2 µg/µL salmon sperm DNA, 50% deionized formamide, 10% dextran sulfate) was dropped on the slides, and the hybridization was performed overnight at 37 °C in a moist chamber containing 2×SSC. The post hybridization wash was carried out with 1×SSC for 5 min at 65 °C. A final wash was performed at room temperature in 4×SSCT for 5 min. Finally, the slides were counterstained with DAPI and mounted in an antifade solution (Vectashield from Vector laboratories) and analyzed in an epifluorescence microscope Olympus BX50 (Olympus Corporation, Ishikawa, Japan).

## Results

### Diploid number, fundamental number and karyotype of *Epalzeorhynchos frenatum*, *Puntigrus partipentazona* and *Scaphognathops bandanensis*

Results have shown that the three cyprinid species have the same diploid number of 2n = 50. Although the three species analyzed share the same 2n, there are differences in the fundamental number (NF) and karyotypes i.e. *E. frenatum*: NF = 78, 18 metacentric (m), 10 submetacentric (sm), 10 subtelocentric (st) and 12 acrocentric (a) chromosomes; *P. partipentazona*: NF = 80, 6m, 24sm, 14st, and 6a chromosomes; *S. bandanensis*: NF = 66, 4m, 12sm, and 34a chromosomes (Fig. 1).

### Chromosome marker of *Epalzeorhynchos frenatum*, *Puntigrus partipentazona* and *Scaphognathops bandanensis*

**Figure 2.**
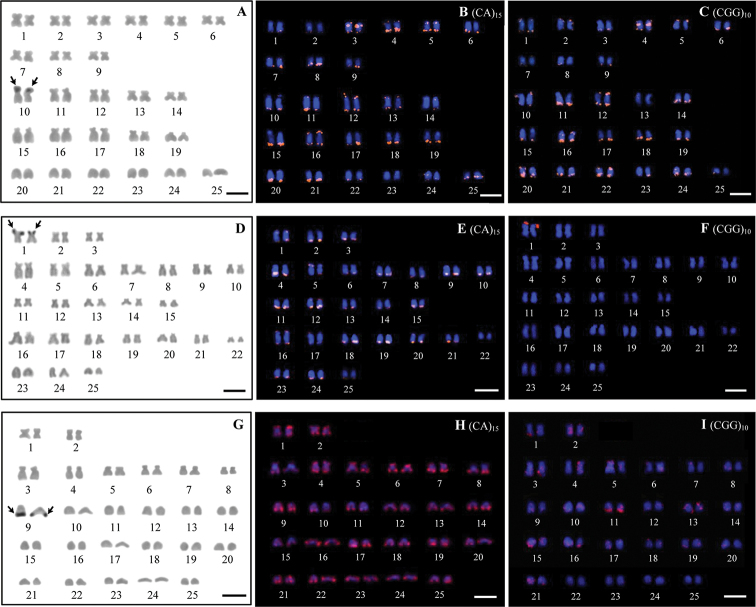
Karyotypes of *Epalzeorhynchos frenatum* (**A–C**), *Puntigrus partipentazona* (**D–F**), *Scaphognathops bandanensis* (**G–I**) by NOR banding and FISH techniques. Arrows indicate NOR-bearing chromosomes. Scale bars: 5 µm.

NOR positive masks were observed at the regions adjacent to the telomere of the short arm of the chromosome pairs 10 (submetacentric) and 1 (metacentric) in *E. frenatum* and *P. partipentazona*, respectively whereas they were revealed at telomeric regions of the long arm of the chromosome pair 9 (acrocentric) in *S. bandanensis* (Fig. 2A, D, G and Table 2).

**Table 2.** Cytogenetic and FISH studies on three Cypinid fishes in Thailand. (2n = diploid chromosome number, NF = fundamental number (number of chromosome arm), m = metacentric, sm = submetacentric, a = acrocentric, st = subtelocentric chromosomes, NOR = nucleolar organizer region).

Species	2n	NF	Chromosome type				Ag-NOR pair (type)	CA_15_ pair	CGG_10_ pair
			m	sm	st	a			
*E. frenatum*	50	84	18	10	10	12	10(sm)	1–13,15–25	1–6,9–12,14–25
*P. partipentazona*	50	94	6	24	14	6	1(m)	1–16, 18–21, 23–25	1
*S. bandanensis*	50	66	4	12	-	34	9(a)	1–25	1, 3–5,9–11, 13, 15–16, 19–21

### Patterns of microsatellite repeats on the genome of *Epalzeorhynchos frenatum*, *Puntigrus partipentazona* and *Scaphognathops bandanensis*

**Figure 3.**
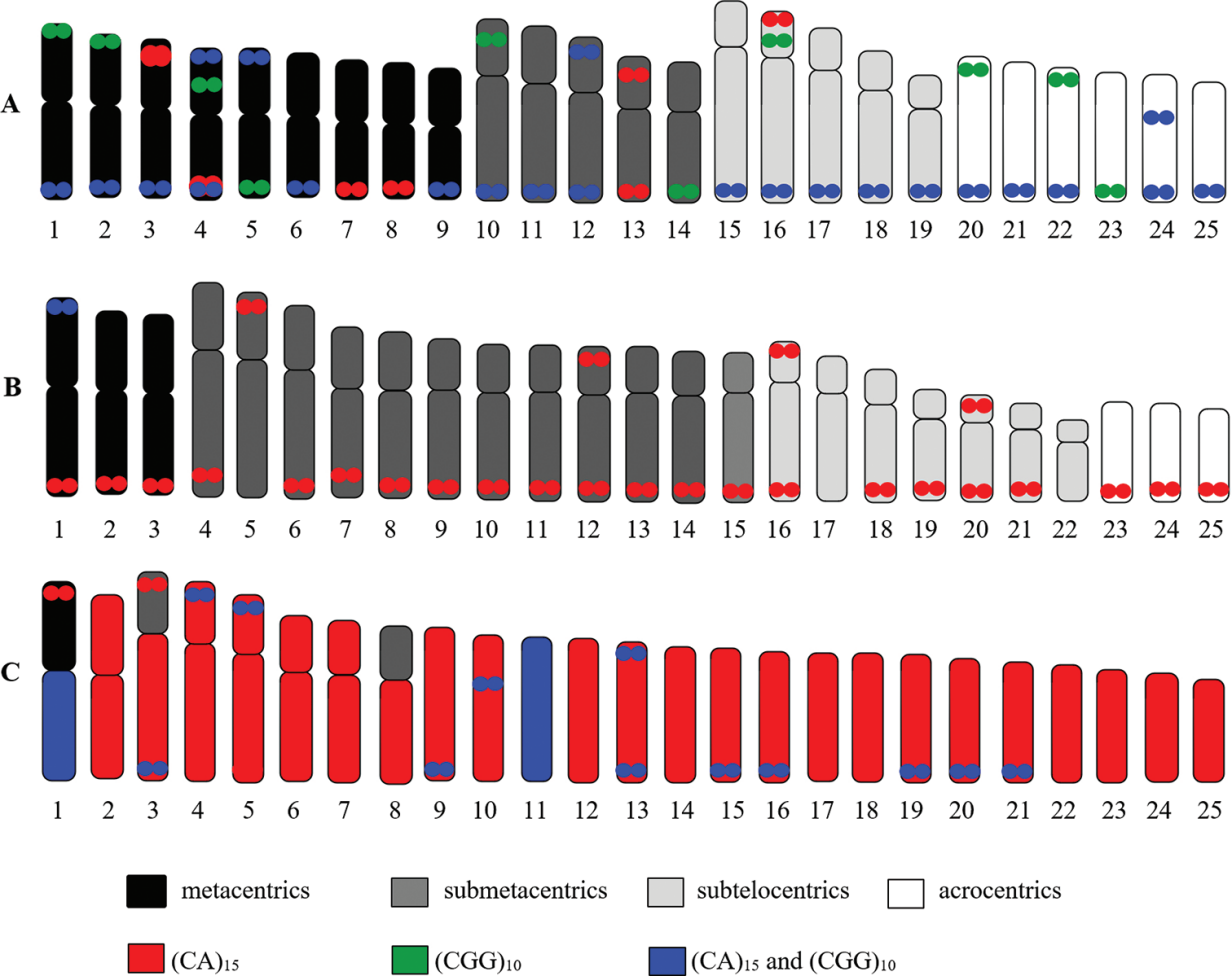
Idiograms represent the (CA)_15_ and (CGG)_10_ mapping on the chromosomes of *Epalzeorhynchos frenatum***A***Puntigrus partipentazona***B***Scaphognathops bandanensis***C**.

The mapping of d(CA)_15_ and d(CGG)_10_ microsatellites shown that hybridization signals are abundantly distributed in telomeric regions of several pairs except d(CA)_15_ repeats in *S. bandanensis*, which are distributed throughout all chromosomes and d(CGG)_10_ repeats in *P. partipentazona* display the high accumulation only in the first chromosome pair. In addition, interstitial signals of d(CA)_15_ and d(CGG)_10_ repeats can be observed at the short arm of the chromosome pairs 3 and 4, respectively in *E. frenatum* (Fig. 2 and Table 2). Figure 3 shows the idiograms representing the patterns of d(CA)_15_ and d(CGG)_10_ microsatellites distributions on the chromosomes of three studied species. Microsatellite d(CGG)_10_ sequences were detected disperse hybridization signals with high accumulation of them at telomeric regions of several chromosomes in *E. frenatum* and *S. bandanensis*. However, it is interesting that the microsatellite d(CGG)_10_ repeats coincide with the NOR positions in *P. partipentazona*.

## Discussion

### Diploid number, fundamental number and karyotype of *Epalzeorhynchos frenatum*, *Puntigrus partipentazona* and *Scaphognathops bandanensis*

The diploid numbers (2n) are same as found in *P. partipentazona* (Taki et al. 1977) and *S. bandanensis* (Donsakul et al. 2007) but there is difference in *E. frenatum* (2n = 48) reported by Magtoon and Donsakul (1993). The 2n in three cypinids studied have the same 2n = 50 as in several species in the subfamily Cyprininae (Arai 2011, Table 1). It seems to be that this subfamily is highly conserved for the 2n. To compare with the previous studies, the NF of *S. bandanensis* is same as the study of Donsakul et al. (2007) whereas ones of *E. frenatum* and *P. partipentazona* differ from the reports of Magtoon and Donsakul (1993) and Taki et al. (1977), respectively. The differences of NFs have cause to differences of karyotypes among these fishes. These differences may be causes from the species-specific variations among populations, and/or misidentification of species or different species due to complex species. Three studied species cannot be observed heteromorphic sex chromosomes between male and female specimens. This phenomenon is same as many species in this family (Arai 2011).

### Chromosome marker of *Epalzeorhynchos frenatum*, *Puntigrus partipentazona* and *Scaphognathops bandanensis*

The determination of nucleolar organizer regions (NORs) for these species was firstly proposed. If these loci are active during the interphase before to mitosis, they can be detected by silver nitrate staining (Howell and Black 1980) since they specifically stain a set of acidic proteins related to ribosomal synthesis process. The single NOR-bearing chromosome pair in the present result is consistent with results from *Barbonymus gonionotus* (Bleeker, 1849) (Khuda-Bukhsh and Das 2007), *Hypsibarbus wetmorei* (Smith, 1931) (Piyapong 1999), *Osteochilus waandersi* (Bleeker, 1853) (Magtoon and Arai 1993) and *Puntius brevis* (Bleeker, 1849) (Nitikulworawong and Khrueanet 2014). This character is common characteristic found in many fish groups as well as vertebrates (Supiwong et al. 2012, 2013). However, some species had two pairs (*Cyclocheilos enoplos* (Bleeker, 1849): Magtoon and Arai 1993), three pairs (*Cyclocheilichthys apogon* (Valenciennes, 1842): Chantapan 2015) and four pairs (*Puntius denisonii* (Day, 1865), *P. semifasciolatus* (Günther, 1868): Nagpure et al. 2004; *P. filamentosus* (Valenciennes, 1844): Nagpure et al. 2003). NORs are chromosomal landmarks that consist of tandemly repeated sequences of ribosomal genes (rRNA). In eukaryotes, each unit is composed of three genes coding for 18S, 5.8S and 28S ribosomal RNA (Sharma et al. 2002). The number and position of the rDNA clusters have been widely used in systematics and phylogenetic reconstructions since these chromosomal characters are often species-specific (Britton-Davidian et al. 2012). Changes in chromosome number and structure can alter the number, and structure of NOR. Structure, number, and morphology of a NOR may be specific to populations, species, and subspecies. Robertsonian translocations (centric fusion) may cause losses of NOR. Studies on NOR variation in numerous organism groups have invariably described changes in the number and location of NORs even in closely related species, suggesting that rDNA clusters are highly mobile components of the genome (Britton-Davidian et al. 2012). Thus, species, which have limited gene exchange due to geographical isolation, have elevated karyotype varieties and NOR variations. The use of NORs in explaining phylogenetic relationships depends on a large extent on the uniformity of this characteristic and on the degree of variety within a taxon (Yüksel and Gaffaroğlu 2008). Normally, most fishes have only one pair of small NORs in a chromosome complement. If some fishes have more than two NORs, it may be caused by the translocation between NOR and another chromosome (Sharma et al. 2002).

### Patterns of microsatellite repeats in the genome of *Epalzeorhynchos frenatum*, *Puntigrus partipentazona* and *Scaphognathops bandanensis*

The patterns of microsatellite d(CA)_15_ in three species in the present study except in *S. bandanensis* are different from the nine species of the Bagridae family including *Hemibagrus filamentus* (Fang & Chaux, 1949), *H. spilopterus* Ng & Rainboth, 1999, *H. wyckii* (Bleeker, 1858), *H. wyckioides* Fang & Chaux, 1949, *Mystus atrifasciatus* Fowler, 1937, *M. multiradiatus* Roberts, 1992, *M. mysticetus* Roberts, 1992, *M. bocourti* (Bleeker, 1864), and *Pseudomystus siamensis* (Regan, 1913) (Supiwong et al. 2013, 2014), *Toxotes chatareus* (Hamilton, 1822) (Supiwong et al. 2017). From the previous and current studies, it may seem that all heterochromatins in fish genomes consist of microsatellites (Cioffi and Bertollo 2012). However, microsatellites have also been found in noncentromeric regions, many of them were located either near or within genes (Rao et al. 2010). This is the same as in the pattern of microsatellite d(CGG)_10_ revealed in *S. bandanensis*.

## Conclusions

The present research is the first report on the NOR -banding and FISH techniques in *E. frenatum*, *P. partipentazona*, *S. bandanensis*. Although all studied species have the same diploid chromosome number (2n = 50) and two NOR-bearing chromosomes, there are differences in the fundamental numbers, numbers of chromosomes with equal sizes, pairs having NORs, and patterns of microsatellites distributions on chromosomes. The NORs can be observed at the regions adjacent to the telomeres of pairs 10, 1 and 9, respectively. The microsatellites are distributed throughout the chromosomes with high accumulations at some positions or all chromosomes which are species-specific characteristics. This result indicated that cytogenetic data can be used for classification in related fish species which have similar morphology.
